# Nurse-family conflict beyond the walls of Iranian homes who have the mechanical ventilation dependent patient: a qualitative research

**Published:** 2015

**Authors:** ST Moradian, K Nourozi, A Ebbadi, HR Khankeh

**Affiliations:** * Department of Nursing, University of Social Welfare and Rehabilitation Sciences, Tehran, Iran,; ** Behavioral Sciences Research Center (BSRC), Nursing Faculty of Baqiyatallah University of Medical Sciences, Tehran, IR Iran

**Keywords:** home care aids, conflict, conflict resolution, communication, nursing

## Abstract

**Rationale.** Home health care (HHC) has been developed more than any other industry in the past decade. Conflict between nurse and family can diminish the aid produced to the home care patients.

**Objective.** This research was guidance to explore the nature of conflicts between the patient’s families and nurses in homes of dangerous care patients, in an Iranian context.

**Methods and results.** Using the qualitative comfortable study system and the purposive sampling, 15 participants including 7 nurses (4 males and 3 females) operating in houses and 8 members of family who had a care receiving mechanical ventilation at house, were interviewed during 2013 and 2014. The main sources of conflict were due to nurse expectations, family belief, and personal qualities of nurses. The team guider tried to prevent the conflict by different measures, but in some samples, the conflict arose. Both members of family and nurses accepted the team leader as the judge. At first, he tried to keep the situation stable and gave some notification to the nurse and some descriptions to the members of family. In some samples, that the family could not adapt to the position and efforts to solve the conflict were unsuccessful, the team relation with the family being cut.

**Conclusion.** Home care situation is prone to conflict due to various factors. The mentioned sources of conflict in home trial were changed from the ones of the hospital. Based on these results appropriate interventions suitable for home conditions should be implemented.

## Introduction

Direction of health services is shifting from the facility based to community based [**1**] because of various changes in the health system such as the increase of more than 300 percent in the elderly population, increasing health care costs, as well as specialist nursing shortage [**1**-**3**]. Recent Technological advancement helped in the provision of the medical services to the complex patients at home [**3**]. Based on these changes, the numbers of complex patients, including the intensive care patients who are being considered for at home are increasing [**4**,**5**]. The transmission of such patients at home, usually occurs in situations when the sick man is already taking different drugs, is disoriented regarding the time and place, has sleep deprivation, malnutrition, as well as stress and fear of death [**6**,**7**]. Also, the client’s families are experiencing stress, anxiety, fear and uncertainty [**8**-**10**].

Some of the ICU patients need continuous care provided by health care professionals at home [**11**]. Nurses are the main care providers at home. Nursing profession is based on a collaborative relationship with colleagues and clients. If two people are viewing the situations from a different perspective, the conflict can compromise their relationship [**12**]. The home care setting is prone to conflict, because the nurses and clients’ families have different values, expectations, perceptions, and backgrounds [**13**,**14**]. Home care nurses are presented to a change of struggles. Someone are from the request for additional visits or family members who expect services more than usual, physicians who do not give orders in a timely manner [**15**].

The conflict with the client’s families is a significant symbol of depersonalization and emotional exhaustion in home care workers [**16**]. Conflict outcomes are not always negative; if the conflict is managed skillfully, it can be a positive experience. If it is not managed well, it can lead to a reduced quality of care and escalate to violence and abuse [**17**-**19**]. 

## Objective

Based on our knowledge, little attention was paid to the interpersonal relationship at home. Also, as it was already mentioned, the home care situation is prone to conflict, so nurses should know how to manage these conditions. However, how this special type of conflict is experienced by nurses and families and how they can manage it, is not being addressed well. Thus, by doing a qualitative approach we wanted to underline the nature of conflicts between the patient’s families and nurses in an Iranian context.

## Materials and methods

To know the nature of relationship between the nurses and the patient’s families and conflicts between them in an Iranian context, the qualitative comfortable study system was used. Qualitative content analysis simplifies data and provides structure and discipline. The content analysis also explores the real meaning behind the raw data [**20**]. In this research, 6 nurses (4 males and 2 females) from the purposive sampling, working at homes, and 6 members of family who had a sick man taking mechanical ventilation at home were examined through 2013 and 2014. Sampling was extended till the data saturation. Nurses who were working at home extra than 2 years and had a thick and rich first knowledge in considering for the ICU sick man at home were included. In order to improve the most change, nurses of males and females, with various work skills and various school levels were chosen. Moreover, members of family from various cultures and socioeconomic levels, giving consideration for the sick man's with various levels of complexity were asked to share. Also, members of family with different durations of involvement in home care were interviewed. 

Semi-structured discussions were used for information gathering and any did from 30 to 70 minutes. Interviews were done in a quiet place, which was offered by study participants. Most of interviews with care of health professions were done in their workplace and discussions with members of family were done at their homes. Someone the nurses was interviewed two times. 

The author of first in this research did the interviews. After many warm-up asks, the discussions were begun by an open-ended question, which they were asked to narrate their experiences in as much details as possible. When needed, the probing and explanatory questions were used for additional clarification of the answers given by participants.

The information investigation was prepared by applying a qualitative comfortable study system. Now following any discussion, it was copied exactly and then it was seen many times in order to obtain a common understanding of the member words. Then, by using the MAXQDA ten application, the information was split in determining factors, later coded, and organized in various subcategories and categories because their relationships and variations. The underlying meanings of the study were expressed in themes because the source of latent content analysis. 

Lincoln and Guba standards since precision and integrity were used in this research [**21**]. The author had a long-term responsibility with the information and research field. Member checking was done by providing a review of the main result of any discussion and at the end results with 3 nurses and 3 family members checked as well. Also the analysis process was done by the approval of 2 members of the study team and was reviewed by 2 outside directors. In cases of difference, the interview was extended until reaching an agreement. Furthermore, the quotes of participants were presented in the finding section.

The Ethical and Study Council of the Social Welfare University and Rehabilitation Sciences in Tehran confirmed this research. The result of the study were clarified for participants. Then, printed learned permission was received from members. Moreover, their permission for recording the discussions was obtained. One participant refused the recording, so, the discussion was done without a voice recorder. The time and place of the interviews was determined by participants and they were able to stop it, if they were exhausted or distressed. 

## Findings

In our study, sixteen interviews with 15 participants were done. 7 nurses played in this research, containing 4 males and 3 females, whose age ranged from 32 to 45 years, their working experience at home was between 3 and 12 years. One of nurses was interviewed two times. In addition, 8 members of family, of whom 5 were male and 3 were female, participated in our study. All of them were close relatives of the clients. Two of them were the patient’s wife, 2 mothers, 2 sons, and 2 were fathers. Their ages ranged between 29 and 65 years and provided care for their patients between 1 month to 5 years (**Table 1**,**2**). The information study explored 552 initial/ open codes. After reviewing and integrating the repetitive codes, 293 initial codes were received. Then codes were clustered in subcategories and categories because similarities and variations, so that, finally, five main themes developed: (a) conflict due to nurses’ expectations; (b) conflict due to the client’s family beliefs; (c) special qualities of nurses; (d) conflict prevention and (e) conflict resolution (**Table 3**).

**Table 1:** Characteristics of the client’s family members

age	gender	Relationship with the patient	Duration of involvement in care	Patient’s diagnosis	Marital status	Duration of interview
65	female	client’s wife	6 months	CVA*	married	30
44	male	son	3 months	CVA	married	45
52	female	mother	8 months	Head trauma	married	38
44	female	mother	18 months	poisoning	married	53
31	male	father	6 months	ALS**	married	52
56	male	son	5 years	Brain ischemia	married	55
44	female	client’s wife	8 months	COPD***	married	48
29	male	father	1 year	ALS	married	39
Abbreviations:						
*Cerebral vascular *accident*, ** Amyotrophic lateral sclerosis, *** Chronic Obstructive Pulmonary Disease						

**Table 2:** Characteristics of professional health care workers

age	gender	position	Experience in home care (year)	Duration of interview (minute)
40	male	team leader	12	70
37	male	nurse	10	45 and 62
45	male	nurse	5	52
33	female	nursing assistant	3	32
41	male	nurse	11	68
36	female	nurse	6	41
32	female	nurse	4	43

**Table 3:** List of codes and categories

category	subcategory	Code (examples)
Conflict due to nurses’ expectations	Mismatch between the job and nurse position	Expecting the nurse to clean the patient’s room
		Not seeing the nurse as a professional staff
		Physical works done by nurses
	Inappropriate behavior of family members	Behavior with the nurse such as a laborer
		Direct order members of family to the nurses
Conflict due to the client’s family expectations	Painful stillness	Expectation of fast healing of the patient
		Lack of improvement is painful
	Expectation of nurse behavior according to family desire	Dissatisfaction of nurse sleeping during the night shift
		Nurse should cook the patient’s food
	Family interference in the treatment	Giving the drugs without prescription by family members
		Interference in the procedures
Special characteristics of nurses	Home care as the second job	Fatigue and exhaustion due to hospital work
		The hospital work is more important for the nurse
	Poor communication skills	Poor management of an irritable family
		Unwillingness to hear critique
Preventing the conflict	Determination of headlines of duties by the team leader	A meeting with the family members on the first day
		Clearing the mutual expectations of nurse and family
	Family education and informing by the team leader	Highlighting the process of coming days
		Explaining the family expectation
	Preparing the nurses	Communication management courses
		Communication management based on family stability
Conflict resolution	Team leader as a judge	Team leader is the reference
		All agree with the team leader
	The customer is always right	Considering the financial benefit of the team
		Giving notification to the nurse
	Notification to the parties	Explaining the situation to the family
		Notifying the families
	Stop working with family	Continuing the care providing to the extent possible
		Cutting the relation in unsuccessful tries

**Conflict due to nurses expectations**

The most nurses working at homes and providing care for intensive care patients are experienced, skilled, and proficient. Because of their background, they expect respect from the client’s family members and tend to have a special condition at their work place. However, the home and its special conditions, sometimes does not agree their beliefs. As a professional person, the nurse expects to manage the care plans with nobody’s interference. In some situations, the family interference is more than what is required and can guide to conflict. 

For model, a nurse told, “what I got during those years was that, the behavior of women with nurses at home is bad. When they want a work to be done by the nurse, they command the nurse. They behave with the nurse, as with an illiterate person who cleans the rooms for them. The nurse is a specialist and provides the special type of care there. This can lead to conflict. Basically, the nurse-family conflict occurs frequently” (Participant 2, a 12 years experienced nurse).

Another nurse says, “The level of the works done at home is not suitable for expert nurses. When we are working in hospital, the nursing assistant cleans the patient; hence, it is hard for us to do it at home. Some families have some expectations such as the nurse should clean the floor. Is it possible that the nurse also prepares the foods? Could the nurse change the bed sheet? However, the nurse does not accept these issues. These can be sources of conflict.” (Participant 3, a 4 years experienced nurse).

**Conflict due to the client’s family beliefs**

The client’s family experiences a high level of stress, fear, and uncertainty due to the client’s complex situation. The family is not ready for the situation in which a complex patient with a team is present at home full time. Therefore, the family member is excitable and unstable. In addition, the family pays money and expects high quality services. The family has the direct responsibility of following up and supervising the treatment and care process. However, they are not close to the domains of the work, standards, as great as the steps of any procedure. As a conclusion of these responsibilities, they have expectations that are sometimes not met. In some examples, the members of family have different ideas regarding the way work should be followed up. Also they are not close to the nursing profession, so the expectations of these two major groups are not matched and may be a source of conflict. Otherwise, there is a close correlation between nurse and family; sometimes the domains of duties being mixed. 

A family member said, “Who’s paying expects the healing and improvement. Our patient’s situation did not change during the past weeks. Stagnation is painful for the client’s family. Our expectations are not met.” (Participant 12 who has been caring for her husband for 8 months).

Extra member told, “We do not have a special organization for home work. All the persons who are here, also work in hospital. The home care is their second job. Someone who is beside the patient should be responsible full time, but most of them have to go to the hospital early in the morning, so they sleep at the patients’ homes.” (Participant 9 who has been caring for his son for 5 years).

**Special characteristics of nurses**

In Iran, nurses almost see the home care as a second job. Therefore, they are using most of their time and energy in the hospital. Because loss of a good communication skill, as great as fatigue and exhaustion, they may experience a bad conversation with the members of family. Another reason is that, most of them operate in the controlled situation of ICU and cannot tolerate the full time appearance of a member of family. Therefore, the appearance of a member of family and the inappropriate communication skills of a nurse could be a source of conflict. 

A family member said, “Every nurse who comes here has a unique style and behavior. Each of them does the suction, gavage, and change position in a special manner. Therefore, we have to adapt to different work styles and behaviors. It made the situation a little hard for us.” (Participant 7 who has been caring for his father for 1 year).

Another family member said, “Most of them work for subsistence and have physical and mental fatigue. Moreover, they are impatient and cannot tolerate critique. They communicate badly. Some of them do not know how to respond to our requests.” (Participant 6 who has been caring for his father for 6 months).

**Preventing the conflict**

In most cases, team members have some conflict prevention strategies. They think that the family is placed in a conflicting and new situation. They are exposed to a situation to which people do not have any previous experience, and, as a conclusion, they do not understand how to manage this new situation. The most teams have a meeting with the family members on the initial day of the transferal of the patient home and offer some explanations about the situation and headlines of the duties. Also the communication rules and mutual expectations are cleared. The teams also have some communication management courses for nurses. Team leaders teach them how to manage the unstable families. They also teach them how to operate in a stable manner so that the family does not abuse them. 

For example, a team leader said,”before transferring the patient home, we have a meeting with our team members. We talk about the patient’s situation, the needed care plan, and also the behavioral situation of the family. Based on these comments we have a communication plan for every family. Also we have a session with the family members in the first hours after the patient’s transfer. We talk to them about their patient’s situation and works that the nurse should do. In addition, their duties are defined. This act could prevent the conflict in most cases.” (Participant 6, an 11 years experienced team leader).

**Conflict resolution**

The difference between the members of the family and the nurse increases gradually and reaches to a limit that cannot be solved between them. Therefore, they search for a reference for judgment between them. Usually, the private team leader is the first reference for the problem solving. The conflict is reported, and the team leader thoroughly assesses the situation. Different problem-solving methods are used. Based on the position, in this step, the team leader decides to practice one of the potential answers. At first, he considers the financial situations of the team and gives the right to the members of the family. Therefore, in this first step he gives some notifications to the nurse. In more complex cases, the team leader gives some reminders to members of the family as great as to the nurse. In severe conflicts, the incompatible person is withdrawn from the care program. The team leader tries to continue the care providing to the extent possible. In some samples, the family cannot adapt to the situation and efforts to resolve the conflict are unsuccessful, in addition, the team similarity with the family will be cut. 

Team guider told, “I try to prevent the conflict. Usually, I talk to the nurse to get shorter, and, after that, I speak to the family members and warn them about their behavior. In most cases, this can prevent severe conflict. For example, one of the nurses was going out of the patients’ room much. The client’s daughter told me he works well, but he is going out of the room much and watches TV. I reported him. The problem was solved.” (Participant 8, a 10 years experienced team leader).

Another nurse said, “The patients’ daughter and mother were intervening very much. We reported the situation to the team leader. He spoke to them. At present, the situation is better.” (Participant 8, a 3 years experienced nurse).

## Discussion

Our findings showed that due to various factors, the home environment is prone to conflict. The conflict could result from the nurse’s expectation, family members’ expectation or associated with the unique aspects of nurses. The main themes or concepts of this research were related to conflict because of the family’s expectations, the nurse’s expectations, unique aspects of nurses, preventing the conflict and conflict resolution. Most of studies about conflict are done in hospital setting and only little attention is spent on the home based conflicts. These studies indicated that poor communication skills is the most critical parts in conflicts between the client’s family and the nurse [**22**,**23**]. Some of these conflicts are also seen in home setting, but it seems that the types of conflicts in home setting are different. In the hospital, the family members are outsiders and have some problems in entering the treatment circumstance, but at home, the nurse is an outsider [**24**]. Hence, he should try to enter the home environment and adopt this new situation. 

The first theme extracted from the data analysis was the conflict due to the nurse’s expectations. As a qualified member of the health team, the nurse has some expectations that may not be achieved at home. One of the reasons for this phenomenon is different between the viewpoint of the nurse and the one of the client’s family. The difference in values, goals and believes could lead to conflict [**25**]. It is possible that the family members are not ready to know the situation well.

The second theme of this research was the unmet expectations of the client’s family members. The client’s family members did not receive any financial support from the insurances and paid all the costs from their pocket. Therefore, they expected high quality care and good outcomes. Sometimes this viewpoint leads to a severe conflict with nurses. Families are anxious about the money and their patient’s outcome. Other studies mention the financial problems as a source of conflict [**22**]. Nurse could give timely and enough information about the cost of attacks and support the family choose between different possible treatments. This intervention could prevent the conflict.

In addition, the family members are not well prepared for this stressful and hard situation. Therefore, maybe they are irritable and prone to conflict. Education is a communication bridge [**22**]. Giving the proper information to the family members about the client’s situation and the treatment process is helpful [**26**]. Also empathy with family members could reduce the anxiety [**27**]. 

Special characteristics of nurses were the significant predictors of conflict. Nurses described the poor conversation arts as a significant factor resulting in conflict. Effective nurse-family relationship is considered central to quality nursing and emotional support [**28**]. Nurses can mitigate the conflict effects by improving their communication skills [**29**]. In Iran, we do not have a comprehensive framework for home care. The most qualified nurses operate at home as a second job. So, they are stressed, frustrated and angry [**30**]. Many of other formal caregivers are inadequate and have weak conversation arts. The studies published in our country show the nurse-family relationship inappropriate and ripe to conflict [**31**,**32**]. Inappropriate relationship could cause stress, temper, lack of confidence, violence and dissatisfaction [**22**].

Poor communication is described as a conflict building factor, but the friendship and calm relationship between nurse and family members could lead to interest [**33**]. So, fulltime professional nurses with good communication skills are needed in our country. Stress is seen in family members, the nurses should see this need and use a collegial relationship instead of coercion [**17**]. 

Conflict prevention is significant in the area of home health care. Before discussing about the intervention for conflict resolution, it is better to prevent it. The home has a different circumstance compared to the hospital and has unique challenges, so, the type of conflict is different. The most current articles suggest the ways of conflict resolution [**12**,**34**]. The prevention of conflict in home care setting is not well addressed in literature. Most of the teams have a meeting with the family members on the first day of transferring the client home in order to specify the headlines of duties. 

Nurses who know the way the conflict is escalated, it is possible to prevent it and improve care providing [**12**]. One of the methods is client centered care that give information to the sick man and participate in decision making [**35**]. This strategy could prevent the conflict.

Despite the preventive strategies, the conflict may happen and lead to critical incidents. Various methods of conflict resolution are used based on cultural diversities. In most references, there are five styles of confrontation with conflict including avoidance, competition, accommodation, compromise and collaboration [**29**]. Any strategy is valuable for its appropriate situation and nurses should learn to judge which strategy is useful for each situation. In this research, the avoidance was the most commonly used strategy. Due to the financial benefit of the team, the nurse managers usually neglect the value and rights of nurses. In this strategy, the person neglects his own goals, values and concerns [**19**]. It is also called a lose-lose strategy and has the worse outcome for nurses. The collaboration is described as the best way for conflict management. Collaboration results in a resolution with desirable outcomes, so, this strategy is the win-win [**36**]. 

**Study limitations**

Because of the cultural barriers, the entrance in the homes for doing the discussions was the strongest factors of this research. The families refused that the researcher enters their home. As the team heads had a familiar association with they considered the family members and in most cases, we required the team administrators to coordinate the time of discussion. 

The other problem was regarding the voice recorder. Some family members did not accept the voice recording. Therefore, one of the interviews was done without sound recorder and in other cases, sound recording was stopped whenever the participants requested. 

## Conclusion

Home care situation is prone to conflict due to various factors. The mentioned sources of conflict in home care are different from the hospital. Because of these results, appropriate interventions suitable for home conditions should be implemented. 

**Acknowledgement**

This study is part of a PhD thesis supported by The Deputy of Research of The University of Social Welfare and Rehabilitation Sciences in Tehran. The writers would like to thank all the nurses and the client’s family members for their service and support in the research.

**Financial Disclosure**

The writers have no struggle of interests.

**Funding/ Support**

This article has been obtained from a thesis analysis plan and was supported by the Deputy of Research, Social Welfare University and Sciences of Rehabilitation, Tehran, Iran.
